# Enriched Environments as a Potential Treatment for Developmental Disorders: A Critical Assessment

**DOI:** 10.3389/fpsyg.2019.00466

**Published:** 2019-03-06

**Authors:** Natalie J. Ball, Eduardo Mercado, Itzel Orduña

**Affiliations:** ^1^Neural and Cognitive Plasticity Laboratory, Department of Psychology, University at Buffalo, The State University of New York, Buffalo, NY, United States; ^2^Department of School and Counseling Psychology, University at Buffalo, The State University of New York, Buffalo, NY, United States

**Keywords:** plasticity, cortical reorganization, learning, cognitive development, animal model

## Abstract

The beneficial effects of enriched environments have been established through a long history of research. Enrichment of the living conditions of captive animals in the form of larger cages, sensory stimulating objects, and opportunities for social interaction and physical exercise, has been shown to reduce emotional reactivity, ameliorate abnormal behaviors, and enhance cognitive functioning. Recently, environmental enrichment research has been extended to humans, in part due to growing interest in its potential therapeutic benefits for children with neurodevelopmental disorders (NDDs). This paper reviews the history of enriched environment research and the use of enriched environments as a developmental intervention in studies of both NDD animal models and children. We argue that while environmental enrichment may sometimes benefit children with NDDs, several methodological factors need to be more closely considered before the efficacy of this approach can be adequately evaluated, including: (i) operationally defining and standardizing enriched environment treatments across studies; (ii) use of control groups and better control over potentially confounding variables; and (iii) a comprehensive theoretical framework capable of predicting when and how environmental enrichment will alter the trajectory of NDDs.

## Introduction

Enrichment of the environment has long been proposed as a treatment or strategy for increasing cognitive ability and well-being, namely in rodents (Cooper and Zubek, 1958; Manosevitz, 1970) and in children in educational contexts (Stoddard and Wellman, 1940; Gruber, 1975). In animal studies, the nature of enrichment varies (Nithianantharajah and Hannan, 2006), but typically involve access to larger, more stimulating environments, with increased opportunities for socialization and voluntary physical activity (van Praag et al., 2000). “Enrichment” generally refers to increases in the variety and/or amount of multisensory stimulation, with the goal being to elicit exploratory behavior (Baroncelli et al., 2010). Enriched environments have been lauded for reducing reactivity to stress and anxiety (Veena et al., 2009; Varman et al., 2012), increasing cognitive function (Arai and Feig, 2011), and enhancing learning and memory mechanisms (van Praag et al., 2000; Arai and Feig, 2011). The impact of enrichment on early development has been studied mainly in comparison to laboratory rodents raised in standard conditions. Recently, environmental enrichment also has been applied as a treatment for neurodevelopmental disorders (NDDs). In this review, we will discuss how enriched environments are thought to affect typically developing animals and humans, will briefly summarize the literature on NDD animal models, and then will evaluate how enriched environments are currently being used in the treatment of NDDs. This review will cover autism spectrum disorder (ASD), attention deficit hyperactivity disorder (ADHD), and Fragile X syndrome (FXS), since more research exists on how enriched environments affect animal models of these disorders, and because environmental enrichment has been proposed as a treatment for these NDDs in children. We will then discuss whether current use of environmental enrichment as a treatment approach is evidence-based as well as potential issues concerning its use.

## Origins of Interest in Enriched Environments

The effects of environmental enrichment are of interest to cognitive scientists because everyday experiences can potentially enhance or inhibit cognitive plasticity and therefore the ability to learn. Cognitive “fitness” may be directly or indirectly related to environmental conditions during development and may be analogous to physical fitness. In the late 19th century, Santiago Ramón y Cajal (1894) realized the potential importance of the environment on the development and function of the brain [for review of Cajal’s work, see DeFelipe (2006)]. The foundation of studies on enriched environments is the work of Donald Hebb (1949), who is credited with discovering the connection between enriched environments and improvements in cognition and behavior. Hebb noticed that the animals he occasionally brought home for his children to play with performed the best in later behavioral tasks. Subsequent enrichment of laboratory housing by other research groups replicated this result. Rosenzweig et al. (1978) defined environmental enrichment as “a combination of complex inanimate and social stimulation” (p. 564). Rosenzweig determined that social grouping of rats was not sufficient to produce enhancements in cognition, and determined that the availability of inanimate objects was key. The general message from this early research seemed to be: for the average lab rat, a stimulating, larger environment with objects, other rats, and exercise, promoted “better” cognitive functioning than the usual small, single-occupancy box containing only bedding.

Worth brief mention here is the use of environmental enrichment in zoos. Enrichment in zoos often differs greatly from what is provided to laboratory animals and has been studied in several different species. Enrichment in zoos includes a variety of methods, such as feeding techniques and the introduction of sensory objects (Renner and Lussier, 2002), aimed at reducing stereotypic behavior by counteracting boredom and at engaging species-specific actions. Instead of a general definition of what must be included in an enriched environment, zoo animal enrichment is dependent on the species and individual animal’s needs, as the goal of this enrichment is solely for the well-being of zoo animals (Mellen and MacPhee, 2001). Because of this individualized approach and the unique aspects of enrichment used in zoos, zoo studies will not be discussed further in this review. However, it should be noted that the goals of the treatments of NDDs may be closer to the goals of enrichment programs in zoos (to counteract individual negative behavioral trajectories), whereas the original goal of environmental enrichment in laboratories was to explore its effects on animals’ brains and their learning and memory abilities.

Rats’ natural environments vary by species. For example, *Rattus rattus* (ship rats) prefer arboreal environments, whereas *Rattus norvegicus* (Norway rats) seldom stray from terrestrial habitats (Foster et al., 2011). Pre-weaned pups will stay in or near the nest and will become more interactive with their environment post-weaning. While it may look as though enriched environments are simply more similar to naturalistic settings for rats, the beneficial effects seen in enriched animals may go beyond those attributable to restoration of typical living conditions in the normally-deprived lab rat (Sale et al., 2016). These benefits may in part be due to the fact that enriched animals are free to explore their environment without fear of predators. In captive animals, there may be a dose-response curve related to enrichment: too little, and boredom occurs; just the right amount, and it encourages creativity and engagement; too much may lead to overstimulation or habituation. Unfortunately, we do not presently know the right amount of enrichment for a species, let alone how individual differences might affect optimal levels of stimulation (Lilley et al., 2017).

In humans, interests in how to change or enrich the environments of children and adults have persisted for centuries in the form of schools, churches, and books. These are attempts to increase the opportunity for social interactions and cognitively stimulating materials, promoting learning in a guided participation context (Rogoff, 2003). “Euthenics” was promoted in the 1940’s as a science aimed at improving the environment with the goal of improving people (Seashore, 1941). This field of research was largely abandoned due to its association with eugenics. Scientific interest in the potential benefits of environmental alteration has recently resurged, but research in this area is in its very early stages.

Use of enriched environments as a developmental intervention has been investigated in laboratory animals as a modulator of developmental trajectories. In lab animals, environmental enrichment improves synaptogenesis (the formation of synapses) and the survival of neurons during early development (van Praag et al., 2000). The impact of enriched environments on development of the visual system has been the most widely studied (reviewed by Sale et al., 2009). For example, enriched environments have been found to increase brain-derived neurotrophic factor (BDNF), which is a protein that promotes neuron growth and maturation. Enriched environments also accelerate development of the inhibitory g-aminobutyric acid (GABA) system in the visual cortex of normal rat pups, which can accelerate visual system development and can occur without requiring any visual input (during either dark-rearing or before eye-opening).

Deprivation can inhibit the development of sensory systems and prolong typical temporal windows of experience-expectant plasticity. In animal models, deprivation of visual experience from birth in rats prolongs the maturation of the visual system, impeding its maturation. When dark-reared rats are housed in enriched environments, they experience normal closure of the critical period, which decreases their susceptibility to monocular deprivation compared to dark-reared rats housed in standard housing (Bartoletti et al., 2004). In this way, enriched environments can compensate for, or counteract, the effects of deprivation on sensory system development. Similarly, enriched environments may be able to ameliorate the effects of social deprivation in the form of maternal separation. Environmental enrichment has been shown to “rescue” neural plasticity and to decrease anxiety by normalizing structural enlargement of the basolateral amygdala and reducing anxiety-like behavior induced by maternal separation (Koe et al., 2016). Whether environmental enrichment is used in deprived or non-deprived animals, it seems to act as an enhancer of development, either to compensate for a previous lack of input or to accelerate “normal” development.

In humans, there has been less structured study of the impacts of enriched environments on development. Much of the work on enriched environments with humans has focused on its potential efficacy in aging populations and cognitive reserve in adults, with the idea being that enrichment can add buffers to the brain’s ability to deal with stress and disease (Barulli and Stern, 2013). In this way, environmental enrichment seems to act as a protective factor for future insults to the brain. Environmental enrichment may provide the increased sensory stimulation needed to recover age-related declines and improve cognitive abilities (Leon and Woo, 2018). In adults, quantity of dendritic spines, postsynaptic thickness, and cortical thickness and weight have been shown to be influenced by environmental enrichment modification (Mohammed et al., 2002), and may encourage neuroplasticity, thereby facilitating recovery from stroke (McDonald et al., 2018). In children, we know much more about the effects of environmental enrichment as a compensatory strategy for the negative impacts of sensory and social deprivation (Bradley et al., 1994; Bakermans-Kranenburg et al., 2008). The use of enriched environments in research with humans, like that with animals, is often complicated by widely varied techniques and therapeutic procedures.

The use of enriched environments early in development is believed to be particularly effective during critical or sensitive periods, when brain plasticity is especially influenced by experience. The commercial industry and some political entities have propagated the idea that accelerating the progression of sensitive periods through early training is beneficial for children. Although animal studies have shown that the length of sensitive periods depends on experience, there is no evidence showing that shortening such periods is advantageous. Products such as Baby Einstein DVDs and classical music CDs, that were once popularized by claims of enhancing cognitive development in children under the age of two, have been largely discredited by the scientific community (Christakis et al., 2004; Pietschnig et al., 2010), and may even be detrimental (Christakis et al., 2004; Zimmerman et al., 2007). The “Mozart effect” was originally reported by Rauscher et al. (1993), who showed that college students who heard a Mozart sonata for 10 min performed significantly better on a spatial reasoning task than after silence or a “relaxation tape.” This effect was of much interest to the scientific community, and the popular media interpreted this finding to mean that listening to Mozart’s music can make children more intelligent. However, many subsequent replications of this study did not support the original findings, and they have since been largely discredited (Pietschnig et al., 2010). These environmental-enrichment products would be considered non-compensatory, because they are meant to enhance typical development.

The benefits of enrichment programs like Head Start has long been a topic of debate, with reports showing evidence for (Ludwig and Phillips, 2008) and against (Aughinbaugh, 2001) long-lasting effects. Head Start was implemented in 1965 as an attempt to prepare poor and underprivileged children for kindergarten by providing social, educational, health and nutritional support, with an emphasis on parent involvement (Hinitz, 2014). Head Start may be considered enriching in that it provides a stimulating, supportive environment for the child. Head Start would arguably be considered a compensatory strategy, meant to counteract any deficits in the experiences of children in at-risk populations. Programs like Head Start are generally viewed as beneficial for at-risk children, but may provide only temporary benefits (Rosenzweig, 2002), paralleling results in animal research (Bennett et al., 1974).

When enriched rats are switched to standard housing, cerebral differences induced by environmental enrichment begin to decrease within weeks, with effects lasting longer when enrichment periods lengthen (Bennett et al., 1974). For example, after 80 days of enriched environment exposure, differences in brain weights remained evident for only 21 days after removal of enrichment. Others have found longer lasting sensori- and neuro-motor (Maegele et al., 2015), memory (Escorihuela et al., 1995; Maegele et al., 2015), and noradrenergic functioning (Escorihuela et al., 1995) effects in rats after enriched-environment exposure.

Recognizing the diminishing effects of the Head Start program after the transition to kindergarten, “booster” programs like the Research-based, Developmentally Informed (REDI) interventions have been created to follow-up with Head Start children in their homes during the kindergarten transition. This program seeks to help parents to teach their children with continued enrichment in the form of books and specialized games and play materials (Bierman et al., 2015). The REDI program has been found to increase academic performance, literacy skills, and other social, emotional, and cognitive skills after the transition into kindergarten (Bierman et al., 2015), particularly for children entering schools with low student achievement (Bierman et al., 2014).

Interestingly, physical exercise can produce some of the same beneficial effects as enriched environments on brains and behavior in humans and other animals (for review, see Hillman et al., 2008). Voluntary wheel running in rodents is a common way to measure the effects of exercise, but because a running wheel is typically part of the rodent’s enriched environment, separating the effects of exercise from that of the overall environment can be tricky (for review, see van Praag et al., 2000). A comparison of enrichment only, running only, and a combination of enrichment and running, revealed that increases in neurogenesis, neural number and survival, and neurotrophin levels were only seen in mice that had access to running (Kobilo et al., 2011). van Praag et al. (1999) found that voluntary running in mice doubled the amount of new cell survival at rates similar to enriched environments and suggested that exercise is sufficient for enhancement of dentate gyrus neurogenesis. Others have argued that physical exercise alone cannot account for all of the effects associated with enriched environments (Nithianantharajah and Hannan, 2009). In children, enriched physical education (physically active play and games designed to be cognitively challenging) has been shown to improve motor coordination and inhibitory control (Pesce et al., 2016). This suggests that deliberate play in a structured physical activity program may provide more benefits than traditional physical exercise programs and may speak to the conditions under which physical activity might be most beneficial.

Studies of enriched environments often focus on gains in function that are thought to be mediated by the effects of increased environmental stimulation on plasticity and brain development (Baroncelli et al., 2010). In laboratory studies of rodents, minimal environmental stimulation was commonplace and often considered the baseline group for the effects associated with enriched environment intervention. However, humans generally live in environments that are already rich in sensory stimulation. Interestingly, where people live may be associated with variations in brain structure. For example, a positive association has been found between living near forests and the integrity of the amygdala, suggesting that geographic location might constitute one aspect of environmental enrichment (Kühn et al., 2017). In the context of NDDs, it is believed that environmental enrichment can compensate for deprivation of sensory/social/motor inputs caused by either an under-stimulating environment and/or by dysfunctional sensory systems. Enriched environments may also accelerate delayed developmental trajectories, thereby helping children to achieve age-typical social skills. In the case of compensatory mechanisms, enriched environments are thought to provide increased quantity and variety of inputs that augment neurobehavioral functioning (Woo and Leon, 2013). Enriched environments are also believed to encourage brain growth more generally (Halperin and Healey, 2011).

Though little is known about the effects of environmental enrichment on human development (Sale et al., 2016), there have been a growing number of studies on the potential use of enriched environments on children with NDDs. Below, we review the literature on the use of developmental animal models and human studies that use enriched environment as a compensatory mechanism for NDDs, focusing specifically on ASD, ADHD, and FXS, as these disorders have been most frequently targeted for treatment with environmental enrichment and studied in animal models.

## Enriched Environment Studies of Ndd Animal Models

Animal models of human behavior make it possible to gather important information about contributing factors and treatments from tightly controlled environments. However, because environmental enrichment methodologies can vary between studies, making comparisons between them is difficult (Nithianantharajah and Hannan, 2006). In mouse and rat models of ASD, chemical and genetic modifications are used to elicit ASD-like symptoms in animals that mimic human ASD symptomology. ASD-model animals may exhibit anxious and stereotypic behavior, abnormal grooming habits, or decreased social behavior; no one model corresponds entirely to human ASD criteria. ADHD animal models often are inbred strains of rats that “naturally” exhibit inattention, hyperactivity, and impulsive behavior (Pamplona et al., 2009) (see Table 1 for comparison of symptoms in humans and animal models of these disorders). The animal models most genetically comparable to an NDD in humans are models of FXS. The key gene anomaly for FXS in humans has been engineered in rodents, leading to hyperactivity and altered patterns of exploration see Supplementary Table S1 for a summary of the animal model and human studies presented in this review.

**Table 1:** Comparison of NDD symptomology for humans and animal models.

	Human (from DSM-5)	Animal models
ASD	-Social-emotional reciprocity deficits -Non-verbal communication deficits -Deficits in developing and maintaining relationships -Repetitive behavior -Inflexibility to change in routine or ritualized behavior -Fixated interests -Hyper/hypo sensitivity to sensory inputs	-Anxiety-like behavior -Deficits in play, communication, and social behavior -Stereotypic behavior -Repetitive grooming behavior
ADHD	-Inattention -Hyperactivity and impulsivity	-Inattention -Hyperactivity and impulsivity
FXS	-Intellectual functioning deficits -Physical features such as narrowing of face, large head, prominent forehead Behavioral, social, and emotional problems associated with ASD -Speech and language deficits	-Social and cognitive deficits -Altered exploratory behavior


### Autism Spectrum Disorder

Mice and rats exposed to valproic acid (VPA) at postnatal day 12.5 have been shown to display ASD-like phenotypes, and thus have been used as animal models of ASD. In VPA-exposed rats, enriched environments in the form of physical exercise, multisensory stimulation, and enriched housing were associated with improved social behavior as well as reduced anxiety-like and repetitive/stereotypic behavior, as compared to VPA-exposed rats reared in standard conditions (Schneider et al., 2006). Enriched housing has been found to increase BDNF both in saline-exposed control and VPA-exposed mice compared to unenriched mice. Environmental enrichment also improved anxiety-like behavior and social and cognitive deficits, as well as hippocampal dendritic spine recuperation in the VPA-exposed group (Yamaguchi et al., 2017). Higher levels of repetitive behavior have been associated with decreased activity in the basal ganglia in a typical deer mouse model, which can be partially remediated using enriched housing. Environmental enrichment resulted in increased neuronal activity, increased dendritic spine density, and decreased repetitive behavior compared to controls raised in standard housing (Bechard et al., 2016).

BTBR mice are an inbred model of ASD (not exposed to chemicals like VPA rats), that show phenotypical behavior typical of children with ASD, such as deficits in social and communicative behaviors. When these mice live in enriched housing, the amount of time they spend engaging in repetitive behavior was reduced, compared to BTBR mice in standard housing, although enrichment did not reduce the rigid quality of behavior (Reynolds et al., 2013). Mice lacking the μ-opioid receptor gene (Oprm1-/-) show social competence and communication deficits, and are thus a proposed model of autism. Environmental enrichment in the form of extra maternal stimulation/care (achieved by adding a lactating female) has been shown to normalize atypical maternal separation responses in pups and increase social motivation in juvenile and adult Oprm1-/- knockouts compared to knockouts reared by only their mother (Garbugino et al., 2016).

Maternal environment has been shown to have effects on development. Adult rodents that are born to high licking-grooming and arched-back nursing (LG-ABN) mothers show a reduced fear response to novel situations and a lower HPA response to stress than those born to low LG-ABN mothers (Caldji et al., 1998). In NDD rodent models, cross-fostering is used to examine the effects of genotype and maternal environment on NDD-related behaviors. In a study by Yang et al. (2007) cross-fostering BTBR mice [born to BTBR mothers, but raised by more sociable C57BL/6J (B6) mothers] did not rescue deficits in play and sociability and was not shown to reduce the high self-grooming of BTBR pups. Enrichment type may be important, since in this particular study, maternal enrichment (i.e., better mothers) did not improve ASD-like symptoms, whereas enriched housing was able to improve certain symptoms in this same mouse model (Reynolds et al., 2013).

In rodent models of ASD, environmental enrichment appears to be more effective in ASD-like symptoms caused by drugs (in VPA-exposed rats), but less effective for inbred strains where symptoms are presumed to come from genetics. Maternal enrichment has varying effects as well, with some improvement in atypical behavior seen when a specific strain is double-mothered, but not when another strain was cross-fostered by a “better” mother.

### Attention Deficit Hyperactivity Disorder

The primary animal model of ADHD is the inbred spontaneously hypertensive rat (SHR) that exhibit ADHD symptomology “naturally” (Pamplona et al., 2009). Multimodal, stimulatory, enriched-environment rearing (post-natal day 21 to 3 months) increased performance on several cognitive tasks in SHRs, suggesting that enhanced environments may be a preventative strategy for coping with deficits in learning and cognitive development associated with ADHD. SHRs and Wistar-Kyoto (WKY) control rats have also been shown to perform better on behavioral tests of hyperactivity and inattention after environmental enrichment, whereas no differences were found on measures of impulsivity (Botanas et al., 2016).

Adult SHRs that were raised by typically active WKY mothers did not differ in locomotor hyperactivity from control SHRs raised by SHR mothers (Howells et al., 2009; Gauthier et al., 2015). In contrast, hyper-social behavior was dependent on mother’s strain, with both SHR and WKY strains raised by SHR mothers showing increased hyper-sociability (Gauthier et al., 2015). Results indicate that some ADHD-like behaviors seen in the SHR strain are genetically determined (Howells et al., 2009), whereas others might be partially dependent on nurturing by SHR dams. As with the ASD double-mothering and cross-fostering studies, evidence on whether enriched mothering is a successful form of environmental enrichment in models of NDDs is mixed. Multimodal enrichment early in life appears to be an effective strategy for preventing certain ADHD-type symptoms like inattention and hyperactivity.

### Fragile X Syndrome

Rat models of FXS include the FMR1-knockout (FMR1-KO) mice, which show cognitive deficits, abnormal immature neuronal morphology, and lack the normal fragile X mental retardation protein (FMRP), that is important for normal cognitive development (Restivo et al., 2005; Oddi et al., 2015). FMR1-KO studies have shown that loss of function of the Fmr1 gene leads to overproduction of transient dendritic spines in the somatosensory cortex, resulting in an increased spine turnover (the formation or elimination of spines), which does not seem to be responsive to sensory modulation (Pan et al., 2010). Thus, lower experience-dependent synaptic tuning appears to take place during circuit development. Environmental enrichment in the form of sensory-enhancement of post-weaned rearing conditions can, however, improve some neural morphology (e.g., immature spines in the visual cortex, reduced basal dendrite lengths) and behavioral issues (e.g., hyperactivity, altered exploration) when compared to controls. This benefit is dependent on α-amino-3-hydroxy-5-methyl-4-isoxazolepropionic acid (AMPA) type I metabotropic glutamate receptor (GluR1) levels (Restivo et al., 2005). Additionally, social enrichment early in life (birth to weaning) has been found to rescue hyperactivity and social and cognitive deficits in FMR1-KO mice, as well as neural morphology into adulthood, with no similar effects seen in wild-type controls (Oddi et al., 2015).

## Enriched Environment Studies in Children With Ndds

The need for evidence-based treatments for NDDs has stimulated research aimed at exploring potential benefits of enriched environments for children, similar to those seen in animal models. Ideally, enriched environments would have lasting benefits long after the treatment has ceased, as continued treatment for NDDs is often costly (Halperin and Healey, 2011). In addition, parents often prefer non-psychopharmacological interventions because traditional medication treatments are rife with side-effects. Recent enriched environment studies of NDDs in children have focused on ASD, ADHD, and FXS.

In the context of NDDs, environmental enrichment has generally been equated with *sensory integration therapy* (SIT), an intervention developed by Ayres (1972). Terminology varies across studies, but SIT, and similar terms such as *sensory integration* (SI) tend to be preferred over the term “environmental enrichment.” A review by Reynolds et al. (2010) suggested that several SIT principles overlap with animal environmental enrichment, such as novelty and sensory experiences in the environment, and active engagement in challenging tasks. SIT is commonly used to treat symptoms of ASD, ADHD, and other developmental delays including deficits in speech and motor function. SIT is assumed to improve dysfunctional sensory processing by tapping into neuroplastic properties through particular forms of sensory stimulation like brushing, swinging, or wearing weighted vests (Lang et al., 2012). A meta-analysis of 30 articles from 1972 to 2013 evaluated the effects of SIT with participants who had, or were at-risk of having, a learning or intellectual disability, ASD, or other diagnosis (Leong et al., 2015). Results showed significant treatment effects when SIT was compared to no treatment, but not when it was compared to alternate forms of intervention. Leong and colleagues concluded that “there are a large number of exploratory and poor quality studies in the area of SI” (p. 201). However, more positive reviews also exist, such as the May-Benson and Koomar (2010) review, which revealed positive outcomes following SIT in a variety of areas, with benefits sustained between 3 and 24 months.

### Autism Spectrum Disorder

Autism spectrum disorder is a complex disorder often associated with behavioral, social, cognitive, motor, sensory, and communication dysfunction. The mechanisms leading to ASD are complex, and are thought to involve a variety of exogenous and endogenous factors, including genetic and epigenetic changes at different stages in development (Rusu et al., 2015). In 2012, Lang and colleagues published a review of 25 studies using SITs, where a total of 217 children with ASD ranging in age from 2 to 12 years (*M* = 5.9 years) were treated with some combination of 10 different types of sensory stimulation. The authors found that only three of these studies suggested that SITs were effective, with eight showing mixed results. They determined that many of the studies (including the three reporting positive results) were methodologically flawed (e.g., lacking control groups, not accounting for confounding variables such as enrollment in other programs/therapies, insufficient sample sizes, etc.). Notably, only 4 of the 25 studies included more than 10 children with ASD.

In a recent triad of studies, Leon and colleagues explored the influence of sensory enrichment in children with autism. In the first study (Woo and Leon, 2013), 13 of 28 males, ages 3–12 years, had daily tactile and olfactory sensory enrichment delivered by parents, as well as music and sensorimotor enrichment exercises over a period of 6 months. The remaining children were assigned to a control group that received standard care. In the experimental group, severity of autism and cognition decreased relative to controls. In the second study, conducted by Woo et al. (2015), 28 of 50 children, ages 3–6, were assigned to one of two experimental groups that received different degrees of sensorimotor enrichment delivered by parents (i.e., “full” or “partial”), as in the previous study. Both experimental groups showed improvements in receptive language and non-verbal cognitive abilities, a decrease in atypical sensory responses, and decreases in autism severity. Although children were grouped based on the severity of their ASD symptoms, because there were no statistically significant differences of severity between the control and treatment group, this separation was collapsed in analysis. Consequently, it is difficult to assess how individual differences might have interacted with treatment efficacy. The third study (Aronoff et al., 2016) involved 1,002 children, 1–18 years old (559 had parent-reported autism diagnoses) and evaluated the effectiveness of sensory enrichment treatment from Mendability, LLC, a paid online service that gives instructions to parents, adapted from the previous study interventions. Based on parent implementation, assessments, execution, and reports of improvement, intention-to-treat analysis showed overall improvements in learning, memory, sensory processing, and other areas after up to 7 months of treatment. Although these studies have all reported symptom reduction and have thus been used to support the claims of consumer products, there are questions remaining as to the efficacy of the interventions. For example, program implementation and assessments were conducted by parents, so variability in implementation, assessment bias, and the confounding variable of increased parental attention are all potential weaknesses of these studies. Given the known issues related to SIT-type program studies (Lang et al., 2012), increased control over experimental design and treatment implementations is required for future studies to determine the true effectiveness of these programs.

Early intervention treatments for ASD have historically included applied behavior analysis (ABA) and social skills training (SST). ABA involves the use of operant conditioning techniques to reduce unwanted behavior (e.g., aggression, self-injury) and to encourage desirable behavior (e.g., following instructions, completing tasks). SST is also a form of behavioral modification program, focused on improving social skills and competence through role playing and practice (Mueser and Bellack, 2007). It is debatable whether these types of treatments qualify as environmental enrichment. However, it should be noted that the treatments share several aspects in common with other “enrichment” programs, such as individualized attention and adult-directed activities in novel environments.

More recent approaches to the treatment of ASD include developmental interventions, which shift attention away from the modification of specific actions and toward understanding and fostering developmental processes that are thought to facilitate the emergence of complex social behavior. Some developmental interventions incorporate aspects of ABA. For example, the Early Start Denver Model (ESDM) is a comprehensive early behavioral intervention for infants and preschool-aged children with ASD that integrates ABA principles with developmental and relationship-based approaches, and has been found to improve cognitive functioning and adaptive behavior in toddlers with ASD (Dawson et al., 2010). The Developmental, Individualized, Relationship-oriented (DIR) model, is a prototypical developmental approach developed by Greenspan and Wieder (2009) that focuses on helping children with autism and other communication disorders improve social reciprocity and functional/pragmatic communication. It seeks to do so by establishing and nurturing relationships that create interactive and affective opportunities for the child to gradually progress through the six developmental levels proposed by Greenspan. A randomized controlled study in which parents were trained to interact with their children with ASD using DIR principles at home indicated higher gains in a measure of functional development and in autism rating scores in the DIR treated group than in the control, standard-care group following a 3-month intervention (Pajareya and Nopmaneejumruslers, 2011). A subsequent, longer (12 months), though uncontrolled study reported similar findings as well as a correlations between children’s developmental gains and their autism rating at baseline, indicating that less severely affected children, benefited from the intervention to a higher extent (Pajareya and Nopmaneejumruslers, 2012). Although developmental interventions typically are not described as involving environmental enrichment, a central element of these programs is their focus on increasing positive, reciprocal and attuned interactions between the caregiver or therapist and the child, which is by nature socially enriching and akin to maternal enrichment in animal studies. In addition, these interventions are likely to provide sensory enrichment as they often use play as a vehicle to interaction and engagement, which likely increase the amount of sensory stimulation experienced by the child.

Theraplay is a play-based developmental intervention developed by Jernberg and Booth in the late 1970’s as a directive, relatively short-term intervention to help children with a variety of problems (Booth and Jernberg, 2009). Theraplay incorporates sensorimotor as well as social enrichment. With a primary focus on improving the attachment relationship between parent and child, the activities involve sensory elements (safe touch, singing, proprioceptive stimulation, taste) and gross motor elements incorporated in the forms of challenging and engaging games. Although Theraplay was not developed exclusively for the treatment of ASD or other NNDs, there is some evidence of treatment effects with this population both in its individual (Hiles Howard et al., 2018) and group (Siu, 2014) modalities.

### Attention Deficit Hyperactivity Disorder

Attention deficit hyperactivity disorder affects approximately 2–13% of preschool children and 3–7% of school-age children, and is associated with inattention, hyperactivity, and impulsivity (Hart et al., 2018). Medication and behavioral interventions are common treatments for ADHD in children. However, many parents of young children are not open to the possibility of medication (Hart et al., 2018), and behavioral interventions can be difficult for families to maintain (Benner-Davis and Heaton, 2007), making these treatments less appealing to parents. Additionally, the effects of these treatments have not been found to be long-term. Neurocognitive approaches, including memory and attention training, are common treatment approaches for ADHD (Betker, 2017). However, focus on these specific domains could be problematic because ADHD is a heterogeneous disorder. Another empirically supported treatment for ADHD involves behavioral parent training (BPT), where parents are taught productive strategies of dealing with their child’s behavior that are meant to reduce stress and improve the parent-child relationship as a way of treating ADHD (for review, see Chronis et al., 2004). Potentially, this type of treatment enriches a child’s environment as it improves social interactions and may improve the home environment.

As in children with ASD, children with ADHD may also be at increased risk of sensory processing deficits. Yochman et al. (2004) found that a high percentage of 4- to 6-year-old children with ADHD fell below lower-bound thresholds (scoring lower than 1.5 SDs below the mean score of the control group) for sensory processing deficits, particularly in sensory modulation, as reported by mothers. SI using fine and gross motor activity has been found to be especially effective in reducing hyperactivity and attentional deficit in school-age children when combined with executive functioning therapy (Salami et al., 2017). Physical activity interventions may be a well-tolerated and beneficial treatment for children and adolescents with ADHD. These interventions may alleviate cognitive, physical, and behavioral symptoms (for review, see Ng et al., 2017), and have been found to increase BDNF (Archer and Kostrzewa, 2012). Indeed, increased physical activity may be a key component of environmental enrichment for children with NDDs^1^, although this possibility has not been widely studied in populations outside of ADHD. Benefits of increased activity have been shown without the other aspects of enriched environments in children with ADHD, but at least one study (Salami et al., 2017) suggests that combination therapy may be most beneficial.

### Fragile X Syndrome

Fragile X syndrome is a NDD characterized by intellectual disability, sensory hypersensitivity, attention deficits, and high incidences of epilepsy and ASD, as well as co-occurring ADHD. It is caused by a mutation of the FMR1 gene on the X chromosome, resulting in reduced production of the FMR1 protein (i.e., FMRP) and abnormalities in brain development. FXS is twice as likely to occur in males, who show increased severity of symptoms in relation to females (Glaser et al., 2003). Targeted drug therapies have been developed, including GABA agonists and mGluR5 antagonists, which have also been suggested for the treatment of ASD (Gürkan and Hagerman, 2012). GABAergic system dysfunction, and its role in synapse and circuit development have been implicated as a contributor to deficits in both FMR1-KO mouse models and FXS patients (for review, see Paluszkiewicz et al., 2011). Despite evidence that environmental enrichment can improve both neural and behavioral issues associated with FXS in animal models (Restivo et al., 2005; Oddi et al., 2015), there have been few studies examining the effects of this intervention in children with FXS.

Differences in sensory processing, particularly in the form of hyper-responsiveness to sensory stimuli, have been reported in children with FXS (Baranek et al., 2002; Rogers et al., 2003). Baranek et al. (2008) conducted a longitudinal study of the sensory processing of male children with FXS and found that between the ages of 9 and 54 months, hyper-responsiveness to sensory stimuli tended to increase, while hypo-responsiveness tended to decrease, although there was considerable within-subject variability. The authors suggested that there are likely sensory “subtypes” among these children, which could differentially affect developmental trajectory and interventions. For example, children with early hypo-responsiveness might be at higher risk of subsequently developing hyper-responsiveness. In infants with FXS, there may be patterns of sensory-motor behavior associated with the FXS phenotype that could be used for developmental screening as early as 9 months of age. For example, in their study of 9–12 month-olds, Baranek et al. (2005) found that object-play scores were negatively correlated with developmental milestones such as the age of walking. Severity of symptoms in FXS may be indicative of underlying mechanisms, which may potentially be associated with differing reactions to enriched environments. There is a need for longitudinal studies that are sensitive to typical and atypical developmental trajectories.

The strong genetic link between FXS and symptomology may lead to a mistaken view that FXS can only be treated with pharmacological treatments (Hall, 2009; Moskowitz and Jones, 2015). However, there is little robust evidence for the effectiveness of pharmacological treatments for FXS (Hall, 2009; Rueda et al., 2009), and research on behavioral, psychosocial, and environmental interventions is lacking (Hall, 2009). There is evidence that genetic and environmental influences can effect cognitive outcomes and adaptive behavior. For example, Dyer-Friedman et al. (2002) found that although the cognitive outcomes for girls with fragile X are predicted most strongly by the mean IQ of their parents (which in this study was used as a proxy for genetic influence), the home environment also accounted for a small proportion of the variance in these outcomes. In boys with fragile X, who tend to be more affected than girls, the genetic contribution to cognitive outcomes is diminished relative to that in girls (as only performance IQs were predicted by mean parental IQ), and the quality of boys’ home environment accounted for more of the variance in their cognitive outcomes than it did for affected girls.

Adaptive behavior has been found to be predicted by home environment (in addition to IQ and age) in boys with fragile X, whereas it is most strongly associated with IQ for girls; FMRP is not associated with adaptive behaviors for girls or boys (Glaser et al., 2003). Evidence from animal models suggests that social enrichment (Oddi et al., 2015) and sensory enrichment (Restivo et al., 2005) improve behavioral and neural anomalies associated with FXS; however, there’s a dearth of enrichment studies in humans with FXS that seems to reflect a presumption that enriched environments cannot counteract genetic abnormalities. Given the shared genetic disruption in FXS, compared to the heterogeneous disorders like ASD and ADHD, studies of the effects of enriched-environment treatments on children with FXS could shed light on how enriched environments affect brain morphology.

## Conclusion

In the study of enriched environments in children with NDDs, there are several things that need to be considered. One major impediment is that there is no agreed upon definition of enriched environments in humans. This omission is problematic because effects attributed to “enriched environments” cannot be conclusively attributed to any one aspect of treatment, and the replication of results is difficult to ascertain when studies use varying programs. Similarly, laboratory animal models use varying enriched environmental conditions, which makes generalization to humans difficult because the treatments are not directly comparable. In animal studies, it is not always clear whether enriched housing, training, physical exercise, or all of these factors underlie the effects seen in combined programs, nor is it clear whether the effects of enrichment are compensatory (i.e., fostering the development of alternative behavioral strategies) or truly curative in nature (Will et al., 2004). For laboratory animals, particularly rodents, the general consensus is that the optimal environment should include sensory, motor, cognitive, and social components, and usually consists of a larger cage, grouped animals, different shaped objects that are changed frequently, and a running wheel (Baroncelli et al., 2010). The translation of these elements into modifications of a child’s environment is less than obvious. Although sensory enrichment may be a component of enriched-environment treatment, SIT and similar therapies may or may not be equivalent to environmental enrichment, despite being referred to as such, and assumptions that the same mechanisms underlie their effects are questionable (Aronoff et al., 2016). Similarly, Head Start may be successful in generating positive outcomes as a result of providing an enriched environment, or due to reasons unrelated to sensory or social enrichment, such as improved nutrition that children receive during the program (Rosenzweig, 2002), or reduced exposure to toxins in the home. Unfortunately, at this point environmental “enrichment” is a relative description, rather than a term for a standard or uniform program.

There are several important differences between enriched-environment treatments for human children and those commonly used for laboratory animals. First, animals are typically housed in their enriched environment, whereas children may only have access to enriched environments at school or during therapy sessions. Human enrichment programs vary more than lab animal enrichment in the protocol and stimuli used, and there is an *ad hoc* approach to many human enrichment programs that tend to focus on one aspect of enrichment; sensory stimulation *or* social interaction *or* physical exercise. It is unknown whether one component is key to the effects seen from enrichment (as has been suggested of physical exercise by van Praag et al., 1999), or whether all enriched environments are created equal. It should also be pointed out that there are difficulties in the human enriched-environment literature concerning the lack of placebo controls and double-blind designs, which have typically been included in laboratory-based studies. When a control group is used in human studies, it often involves children who are enrolled in another type of therapy or intervention program (such is the case with many studies on Head Start, Rosenzweig, 2002; and SIT, Lang et al., 2012). In addition, animals experiencing enriched environments in the laboratory are typically able to explore and interact with their environment without fear from predators and other risks associated with life in the wild (Sale et al., 2016), while children are not necessarily exempt from fear and/or stress in their broader environment during their exposure to enrichment programs.

Another aspect to consider is the timing of enriched-environment interventions. In deprivation studies, earlier intervention seems to result in better outcomes for institutionalized children (Bakermans-Kranenburg et al., 2008). For children at risk for NDDs, such as preterm infants, it has been proposed that environmental enrichment (relative to standard conditions in hospitals) should begin as early as possible, potentially while children are still in the Neonatal Intensive Care Unit (Inguaggiato et al., 2017). Some researchers suggest that there are early time windows for susceptibility of impaired synaptic phenotypes in NDDs, and that knowledge about these periods can be useful in early therapeutic treatment (Meredith et al., 2012). For example, synaptic maturation delays, such as those found in FMR1-KO mouse cortex and hippocampus due to lack of FMRP during critical refinement periods (beginning at P7), could underlie later impairments in circuitry and serve as a biomarker for early diagnosis. Though later enrichment can partially rescue some impairment in FMR1-KO mice (Restivo et al., 2005), earlier treatments designed to prevent the development of aberrant pathways would be ideal (Meredith et al., 2012). Unfortunately, diagnosis of NDDs in humans is often made months or years after birth, which may miss sensitive plasticity periods. For example, over half of children with ASD are not diagnosed until after their fifth birthday (Pringle et al., 2012), although parents report awareness of problems in their child’s development by 18 months of age (Howlin and Asgharian, 1999).

Another issue with implementation of environmental enrichment as a potential therapeutic intervention is that there are often large individual differences in how children respond to treatments. Complex disorders develop from varieties of endophenotypes and the symptomology and behaviors associated with a particular disorder can also vary from person to person. In animal models, this is considered less of an issue because all mice share genetic markers and share environments with other animals in their group. One way to address this issue may be to incorporate knowledge of the developmental trajectories associated with specific NDDs. The use of developmental trajectories in understanding developmental disorders can help us understand the underlying causal mechanisms (Thomas et al., 2009). For example, along with heterogeneous phenotypes, the developmental trajectory pathways of autism may also be varied. Fountain et al. (2012) identified six trajectory groups with differing symptom trajectories correlated with socioeconomic factors.

Neurodevelopmental trajectories of ADHD have been evaluated using neuroimaging and neuropsychological studies (for review, see Shaw et al., 2010; Halperin and Healey, 2011). ADHD remission with age has been associated with a normalization of initial brain network delays, and persistence into adolescence may reflect a brain development trajectory that is more abnormal (Shaw et al., 2010). For example, Shaw et al. (2007) found a delay in the development of cortical thickness of approximately 3 years for children with ADHD, particularly in the prefrontal regions. The authors suggested that this may cause a delay of normal maturation into adolescence.

Gene-environment interactions mediating plasticity in complex NDDs are also important to consider. For example, the epigenetic regulator Methyl-CpG-binding protein 2 (MeCP2) has been found to be reduced in expression in individuals with autism, Down syndrome, ADHD, and several other disorders (Nagarajan et al., 2006). Future work should assess whether epigenetic changes occur as a result of environmental enrichment, which may address concerns that enriched-environment benefits may only be temporary. For example, in mice, environmental enrichment can prevent epigenetic changes associated with stress and inflammation in aging mice (Griñan-Ferré et al., 2016), and can affect DNA methylation in the hippocampus around puberty (Zhang et al., 2018). If similar epigenetic changes were found in NDD animal models or children associated with environmental enrichment, we could more definitively say that environmental enrichment has lasting effects.

Enriched environments are popularly implemented as a treatment for several NDDs in children. There is evidence from animal models of ASD, ADHD, and FXS that environmental enrichment could be therapeutic, but generalizability to humans is difficult as the mechanisms that determine which treatments will be beneficial have yet to be identified. Recent interest in enriched environments as a feasible alternative to medications has resulted in a few studies on children. However, there are discrepancies in these approaches as well. A framework for predicting when and how specific treatments will change NDD trajectories is needed. Future work should take into consideration the timing of such interventions, heterogeneity in these complex disorders, and the developmental trajectories of specific NDDs.

## Author Contributions

NB, EM, and IO contributed to the content of this manuscript.

## Conflict of Interest Statement

The authors declare that the research was conducted in the absence of any commercial or financial relationships that could be construed as a potential conflict of interest.

## Footnotes

1 Since there is no set definition of what an enriched environment looks like for a child with an NDD, there are many programs or interventions that could be considered enriching. Using the traditional laboratory animal definition of an enriched environment, one might say that in order to qualify, the program must expose children to large, stimulating environments, with opportunities to play with other children and provide opportunities for voluntary physical exercise (van Praag et al., 2000). Without further delineation, a typical preschool or elementary school classroom might be considered an enriched environment using these criteria.

## References

[B1] Arai J. A. Feig L. A. (2011). Long-lasting and transgenerational effects of an environmental enrichment on memory formation. *Brain Res. Bull.* 85 30–35. 10.1016/j.brainresbull.2010.11.003 21078373 PMC3070197

[B2] Archer T. Kostrzewa R. (2012). Physical exercise alleviates ADHD symptoms: regional deficits and development trajectory. *Neurotox. Res.* 21 195–209. 10.1007/s12640-011-9260-0 21850535

[B3] Aronoff E. Hillyer R. Leon M. (2016). Environmental enrichment therapy for autism: outcomes with increased access. *Neural Plast.* 2016:2734915. 10.1155/2016/2734915 27721995 PMC5046013

[B4] Aughinbaugh A. (2001). Does Head Start yield long-term benefits? *J. Hum. Resour.* 36 641–665. 10.2307/3069637 26802112

[B5] Ayres A. J. (1972). *Sensory Integration and Learning Disorders.* Los Angeles, CA: Western Psychological Services.

[B6] Bakermans-Kranenburg M. J. Van Ijzendoorn M. H. Juffer F. (2008). Earlier is better: a meta-analysis of 70 years of intervention improving cognitive development in institutionalized children. *Monogr. Soc. Res. Child Dev.* 73 279–293. 10.1111/j.1540-5834.2008.00498.x 19121021

[B7] Baranek G. T. Chin Y. H. Hess L. M. Yankee J. G. Hatton D. D. Hooper S. R. (2002). Sensory processing correlates of occupational performance in children with fragile X syndrome: preliminary findings. *Am. J. Occup. Ther.* 56 538–546. 10.5014/ajot.56.5.538 12269508

[B8] Baranek G. T. Danko C. D. Skinner M. L. Bailey D. B. Jr. Hatton D. D. Roberts J. E. (2005). Video analysis of sensory-motor features in infants with fragile X syndrome at 9-12 months of age. *J. Autism Dev. Disord.* 35 645–656. 10.1007/s10803-005-0008-7 16172809

[B9] Baranek G. T. Roberts J. E. David F. J. Sideris J. Mirrett P. L. Hatton D. D. (2008). Developmental trajectories and correlates of sensory processing in young boys with fragile X syndrome. *Phys. Occup. Ther. Pediatr.* 28 79–98. 10.1300/J006v28n01_06 18399048

[B10] Baroncelli L. Braschi C. Spolidoro M. Begenisic T. Sale A. Maffei L. (2010). Nurturing brain plasticity: impact of environmental enrichment. *Cell Death Differ.* 17 1092–1103. 10.1038/cdd.2009.193 20019745

[B11] Bartoletti A. Medini P. Berardi N. Maffei L. (2004). Environmental enrichment prevents effects of dark-rearing in the rat visual cortex. *Nat. Neurosci.* 7 215–216. 10.1038/nn1201 14966527

[B12] Barulli D. Stern Y. (2013). Efficiency, capacity, compensation, maintenance, plasticity: emerging concepts in cognitive reserve. *Trends Cogn. Sci.* 17 502–509. 10.1016/j.tics.2013.08.012 24018144 PMC3840716

[B13] Bechard A. R. Cacodcar N. King M. A. Lewis M. H. (2016). How does environmental enrichment reduce repetitive motor behaviors? Neuronal activation and dendritic morphology in the indirect basal ganglia pathway of a mouse model. *Behavioural Brain Research* 299 122–131. 10.1016/j.bbr.2015.11.029 26620495 PMC4696878

[B14] Benner-Davis S. Heaton P. C. (2007). Attention Deficit and Hyperactivity Disorder: controversies of diagnosis and safety of pharmacological and nonpharmacological treatment. *Curr. Drug Saf.* 2 33–42. 10.2174/157488607779315444 18690948

[B15] Bennett E. L. Rosenzweig M. R. Diamond M. C. Morimoto H. Hebert M. (1974). Effects of successive environments on brain measures. *Physiol. Behav.* 12 621–631. 10.1016/0031-9384(74)90212-1 4824387

[B16] Betker C. (2017). Environmental strategies for managing attention deficit hyperactivity disorder. *J. Child. Dev. Disord.* 3:24 10.4172/2472-1786.100062

[B17] Bierman K. L. Nix R. L. Heinrichs B. S. Domitrovich C. E. Gest S. D. Welsh J. A. (2014). Effects of Head Start REDI on children’s outcomes 1 year later in different kindergarten contexts. *Child Dev.* 85 140–159. 10.1111/cdev.12117 23647355 PMC3740043

[B18] Bierman K. L. Welsh J. A. Heinrichs B. S. Nix R. L. Mathis E. T. (2015). Helping Head Start parents promote their children’s kindergarten adjustment: the Research-based Developmentally Informed Parent Program. *Child Dev.* 86 1877–1891. 10.1111/cdev.12448 26494108 PMC4626262

[B19] Booth P. B. Jernberg A. M. (2009). *Theraplay: Helping Parents and Children Build Better Relationships Through Attachment-Based Play.* New York, NY: John Wiley and Sons.

[B20] Botanas C. J. Lee H. De La Peña J. B. Dela Peña I. J. Woo T. Kim H. J. (2016). Rearing in an enriched environment attenuated hyperactivity and inattention in the Spontaneously Hypertensive Rats, an animal model of Attention-Deficit Hyperactivity Disorder. *Physiol. Behav.* 155 30–37. 10.1016/j.physbeh.2015.11.035 26656767

[B21] Bradley R. H. Whiteside L. Mundfrom D. J. Casey P. H. Kelleher K. J. Pope S. K. (1994). Early indications of resilience and their relation to experiences in the home environments of low birthweight, premature children living in poverty. *Child Dev.* 65 346–360. 10.2307/1131388 8013226

[B22] Cajal S. R. Y. (1894). Consideraciones generales sobre la morfología de la célula nerviosa. *La Vet. Espaǹola* 37 289–291.

[B23] Caldji C. Tannenbaum B. Sharma S. Francis D. Plotsky P. M. Meaney M. J. (1998). Maternal care during infancy regulates the development of neural systems mediating the expression of fearfulness in the rat. *Proc. Natl. Acad. Sci. U.S.A.* 95 5335–5340. 10.1073/pnas.95.9.5335 9560276 PMC20261

[B24] Christakis D. A. Zimmerman F. J. Digiuseppe D. L. Mccarty C. A. (2004). Early television exposure and subsequent attentional problems in children. *Pediatrics* 113 708–713. 10.1542/peds.113.4.708 15060216

[B25] Chronis A. M. Chacko A. Fabiano G. A. Wymbs B. T. Pelham WE Jr (2004). Enhancements to the behavioral parent training paradigm for families of children with ADHD: review and future directions. *Clin. Child Fam. Psychol. Rev.* 7 1–27. 10.1023/B:CCFP.0000020190.60808.a4 15119686

[B26] Cooper R. M. Zubek J. P. (1958). Effects of enriched and restricted early environments on the learning ability of bright and dull rats. *Can. J. Psychol.* 12 159–164. 10.1037/h0083747 13573245

[B27] Dawson G. Rogers S. Munson J. Smith M. Winter J. Greenson J. (2010). Randomized, controlled trial of an intervention for toddlers with autism: the Early Start Denver Model. *Pediatrics* 125 e17–e23. 10.1542/peds.2009-0958 19948568 PMC4951085

[B28] DeFelipe J. (2006). Brain plasticity and mental processes: Cajal again. *Nat. Rev. Neurosci.* 7 811–817. 10.1038/nrn2005 16988656

[B29] Dyer-Friedman J. Glaser B. Hessl D. Johnston C. Huffman L. C. Taylor A. (2002). Genetic and environmental influences on the cognitive outcomes of children with fragile X syndrome. *J. Am. Acad. Child Adolesc. Psychiatry* 41 237–244. 10.1097/00004583-200203000-00002 11886017

[B30] Escorihuela R. M. Fernández-Teruel A. Tobeña A. Vivas N. M. Mármol F. Badia A. (1995). Early environmental stimulation produces long-lasting changes on ß-adrenoceptor transduction system. *Neurobiol. Learn. Mem.* 64 49–57. 10.1006/nlme.1995.1043 7582812

[B31] Foster S. King C. Patty B. Miller S. (2011). Tree-climbing capabilities of Norway and ship rats. *N. Z. J. Zool.* 38 285–296. 10.1080/03014223.2011.599400

[B32] Fountain C. Winter A. S. Bearman P. S. (2012). Six developmental trajectories characterize children with autism. *Pediatrics* 129 e1112–e1120. 10.1542/peds.2011-1601 22473372 PMC3340586

[B33] Garbugino L. Centofante E. D’amato F. R. (2016). Early social enrichment improves social motivation and skills in a monogenic mouse model of autism, the Oprm1 (-/-) mouse. *Neural Plasticity* 2016:5346161. 10.1155/2016/5346161 27274875 PMC4870371

[B34] Gauthier A. C. Deangeli N. E. Bucci D. J. (2015). Cross-fostering differentially affects ADHD-related behaviors in spontaneously hypertensive rats. *Dev. Psychobiol.* 57 226–236. 10.1002/dev.21286 25647439 PMC4336222

[B35] Glaser B. Hessl D. Dyer-Friedman J. Johnston C. Wisbeck J. Taylor A. (2003). Biological and environmental contributions to adaptive behavior in fragile X syndrome. *Am. J. Med. Genet. A* 117A, 21–29. 10.1002/ajmg.a.10549 12548736

[B36] Greenspan S. I. Wieder S. (2009). *Engaging Autism: Using the Floortime Approach to Help Children Relate, Communicate, and Think.* Cambridge, MA: Da Capo Press.

[B37] Griñan-Ferré C. Puigoriol-Illamola D. Palomera-Ávalos V. Pérez-Cáceres D. Companys-Alemany J. Camins A. (2016). Environmental enrichment modified epigenetic mechanisms in SAMP8 mouse hippocampus by reducing oxidative stress and inflammaging and achieving neuroprotection. *Front. Aging Neurosci.* 8:241. 10.3389/fnagi.2016.00241 27803663 PMC5067530

[B38] Gruber J. J. (1975). Effects of enriched academic environment on scholastic achievement of culturally deprived pupils. *Am. Correct. Ther. J.* 29 47–50. 1136923

[B39] Gürkan C. K. Hagerman R. J. (2012). Targeted treatments in autism and fragile x syndrome. *Res. Autism Spectr. Disord.* 6 1311–1320. 10.1016/j.rasd.2012.05.007 23162607 PMC3498468

[B40] Hall S. S. (2009). Treatments for fragile X syndrome: a closer look at the data. *Dev. Disabil. Res. Rev.* 15 353–360. 10.1002/ddrr.78 20014373 PMC2898566

[B41] Halperin J. M. Healey D. M. (2011). The influences of environmental enrichment, cognitive enhancement, and physical exercise on brain development: can we alter the developmental trajectory of ADHD? *Neurosci. Biobehav. Rev.* 35 621–634. 10.1016/j.neubiorev.2010.07.006 20691725 PMC3008505

[B42] Hart K. C. Ros R. Gonzalez V. Graziano P. A. (2018). Parent perceptions of medication treatment for preschool children with ADHD. *Child Psychiatry Hum. Dev.* 49 155–162. 10.1007/s10578-017-0737-9 28646328

[B43] Hebb D. O. (1949). *The Organization of Behaviour.* New York, NY: Wiley.

[B44] Hiles Howard A. R. Lindaman S. Copeland R. Cross D. R. (2018). Theraplay impact on parents and children with autism spectrum disorder: improvements in affect, join attention, and social cooperation. *Int. J. Play Ther.* 27 56–68. 10.1037/pla0000056

[B45] Hillman C. H. Erickson K. I. Kramer A. F. (2008). Be smart, exercise your heart: exercise effects on brain and cognition. *Nat. Rev. Neurosci.* 9 58–65. 10.1038/nrn2298 18094706

[B46] Hinitz B. S. F. (2014). Head Start. *Young Child.* 69 94–97.

[B47] Howells F. M. Bindewald L. Russell V. A. (2009). Cross-fostering does not alter the neurochemistry or behavior of spontaneously hypertensive rats. *Behav. Brain Funct.* 5:24. 10.1186/1744-9081-5-24 19549323 PMC2711096

[B48] Howlin P. Asgharian A. (1999). The diagnosis of autism and Asperger syndrome: findings from a survey of 770 families. *Dev. Med. Child Neurol.* 41 834–839. 10.1017/S0012162299001656 10619282

[B49] Inguaggiato E. Sgandurra G. Cioni G. (2017). Brain plasticity and early development: implications for early intervention in neurodevelopmental disorders. *Neuropsychiatr. L’enfance de l’Adolesc.* 65 299–306. 10.1016/j.neurenf.2017.03.009

[B50] Kobilo T. Liu Q.-R. Gandhi K. Mughal M. Shaham Y. Van Praag H. (2011). Running is the neurogenic and neurotrophic stimulus in environmental enrichment. *Learn. Mem.* 18 605–609. 10.1016/j.bbr.2013.02.018 21878528 PMC3166785

[B51] Koe A. S. Ashokan A. Mitra R. (2016). Short environmental enrichment in adulthood reverses anxiety and basolateral amygdala hypertrophy induced by maternal separation. *Transl. Psychiatry* 6 e729–e729. 10.1038/tp.2015.217 26836417 PMC4872421

[B52] Kühn S. Düzel S. Eibich P. Krekel C. Wüstemann H. Kolbe J. (2017). In search of features that constitute an ”enriched environment” in humans: associations between geographical properties and brain structure. *Sci. Rep.* 7 11920–11920. 10.1038/s41598-017-12046-7 28931835 PMC5607225

[B53] Lang R. O’reilly M. Healy O. Rispoli M. Lydon H. Streusand W. (2012). Sensory integration therapy for autism spectrum disorders: a systematic review. *Res. Autism Spectr. Disord.* 6 1004–1018. 10.1016/j.rasd.2012.01.006

[B54] Leon M. Woo C. (2018). Environmental enrichment and successful aging. *Front. Behav. Neurosci.* 12:155. 10.3389/fnbeh.2018.00155 30083097 PMC6065351

[B55] Leong H. Carter M. Stephenson J. (2015). Meta-analysis of research on sensory integration therapy for individuals with developmental and learning disabilities. *J. Dev. Phys. Disabil.* 27 183–206. 10.1016/j.ridd.2015.09.022 26476485

[B56] Lilley M. K. Kuczaj S. A. Yeater D. B. (2017). “Individual differences in nonhuman animals: examining boredom, curiosity, and creativity,” in *Personality in Nonhuman Animals*, eds Vonk J. Weiss A. Kuczaj S. A. (Cham: Springer International Publishing), 257–275.

[B57] Ludwig J. Phillips D. A. (2008). Long-term effects of Head Start on low-income children. *Ann. N. Y. Acad. Sci.* 1136 257–268. 10.1196/annals.1425.005 17954676

[B58] Maegele M. Braun M. Wafaisade A. Schäfer N. Lippert-Gruener M. Kreipke C. (2015). Long-term effects of enriched environment on neurofunctional outcome and CNS lesion volume after traumatic brain injury in rats. *Physiol. Res.* 64 129–145. 25194132 10.33549/physiolres.932664

[B59] Manosevitz M. (1970). Early environmental enrichment and mouse behavior. *J. Comp. Physiol. Psychol.* 71 459–466. 10.1037/h0029141

[B60] May-Benson T. A. Koomar J. A. (2010). Systematic review of the research evidence examining the effectiveness of interventions using a sensory integrative approach for children. *Am. J. Occup. Ther.* 64 403–414. 10.5014/ajot.2010.09071 20608272

[B61] McDonald M. W. Hayward K. S. Rosbergen I. C. M. Jeffers M. S. Corbett D. (2018). Is environmental enrichment ready for clinical application in human post-stroke rehabilitation? *Front. Behav. Neurosci.* 12:135. 10.3389/fnbeh.2018.00135 30050416 PMC6050361

[B62] Mellen J. MacPhee M. S. (2001). Philosophy of environmental enrichment: past, present, and future. *Zoo Biol.* 20 211–226. 10.1002/zoo.1021

[B63] Meredith R. M. Dawitz J. Kramvis I. (2012). Sensitive time-windows for susceptibility in neurodevelopmental disorders. *Trends Neurosci.* 35 335–344. 10.1016/j.tins.2012.03.005 22542246

[B64] Mohammed A. H. Zhu S. W. Darmopil S. Hjerling-Leﬄer J. Ernfors P. Winblad B. (2002). Environmental enrichment and the brain. *Prog. Brain Res.* 138 109–133. 10.1016/S0079-6123(02)38074-9 12432766

[B65] Moskowitz L. J. Jones E. A. (2015). Uncovering the evidence for behavioral interventions with individuals with fragile X syndrome: a systematic review. *Res. Dev. Disabil.* 38 223–241. 10.1016/j.ridd.2014.12.011 25575286

[B66] Mueser K. T. Bellack A. S. (2007). Social skills training: alive and well? *J. Ment. Health* 16 549–552. 10.1080/09638230701494951

[B67] Nagarajan R. P. Hogart A. R. Gwye Y. Martin M. R. Lasalle J. M. (2006). Reduced MeCP2 expression is frequent in autism frontal cortex and correlates with aberrant MECP2 promoter methylation. *Epigenetics* 1 e1–e11. 10.4161/epi.1.4.3514 17486179 PMC1866172

[B68] Ng Q. X. Ho C. Y. X. Chan H. W. Yong B. Z. J. Yeo W.-S. (2017). Managing childhood and adolescent attention-deficit/hyperactivity disorder (ADHD) with exercise: a systematic review. *Complement. Ther. Med.* 34 123–128. 10.1016/j.ctim.2017.08.018 28917364

[B69] Nithianantharajah J. Hannan A. J. (2006). Enriched environments, experience-dependent plasticity and disorders of the nervous system. *Nat. Rev. Neurosci.* 7 697–709. 10.1038/nrn1970 16924259

[B70] Nithianantharajah J. Hannan A. J. (2009). The neurobiology of brain and cognitive reserve: mental and physical activity as modulators of brain disorders. *Prog. Neurobiol.* 89 369–382. 10.1016/j.pneurobio.2009.10.001 19819293

[B71] Oddi D. Subashi E. Middei S. Bellocchio L. Lemaire-Mayo V. Guzmán M. (2015). Early social enrichment rescues adult behavioral and brain abnormalities in a mouse model of fragile X syndrome. *Neuropsychopharmacology* 40 1113–1122. 10.1038/npp.2014.291 25348604 PMC4367453

[B72] Pajareya K. Nopmaneejumruslers K. (2011). A pilot randomized controlled trial of DIR/Floortime parent training intervention for pre-school children with autistic spectrum disorders. *Autism* 15 563–577. 10.1177/1362361310386502 21690083

[B73] Pajareya K. Nopmaneejumruslers K. (2012). A one-year prospective follow-up study of a DIR/Floortime parent training intervention for pre-school children with autistic spectrum disorders. *J. Med. Assoc. Thai.* 95 1184–1193. 23140036

[B74] Paluszkiewicz S. M. Martin B. S. Huntsman M. M. (2011). Fragile X syndrome: the GABAergic system and circuit dysfunction. *Dev. Neurosci.* 33 349–364. 10.1159/000329420 21934270 PMC3254035

[B75] Pamplona F. A. Pandolfo P. Savoldi R. Prediger R. D. S. Takahashi R. N. (2009). Environmental enrichment improves cognitive deficits in Spontaneously Hypertensive Rats (SHR): relevance for Attention Deficit/Hyperactivity Disorder (ADHD). *Progr. Neuro Psychopharmacol. Biol. Psychiatry* 33 1153–1160. 10.1016/j.pnpbp.2009.06.012 19549550

[B76] Pan F. Aldridge G. M. Greenough W. T. Gan W.-B. (2010). Dendritic spine instability and insensitivity to modulation by sensory experience in a mouse model of fragile X syndrome. *Proc. Natl. Acad. Sci. U.S.A.* 107 17768–17773. 10.1073/pnas.1012496107 20861447 PMC2955121

[B77] Pesce C. Masci I. Marchetti R. Vazou S. Sääkslahti A. Tomporowski P. D. (2016). Deliberate play and preparation jointly benefit motor and cognitive development: mediated and moderated effects. *Front. Psychol.* 7:349. 10.3389/fpsyg.2016.00349 27014155 PMC4786558

[B78] Pietschnig J. Voracek M. Formann A. K. (2010). Mozart effect–Shmozart effect: a meta-analysis. *Intelligence* 38 314–323. 10.1016/j.intell.2010.03.001

[B79] Pringle B. Colpe L. J. Blumberg S. J. Avila R. M. Kogan M. D. (2012). *Diagnostic History and Treatment of School-Aged Children with Autism Spectrum Disorder and Special Health Care Needs.* Hyattsville, MD: National Center for Health Statistics. 23050521

[B80] Rauscher F. H. Shaw G. L. Ky K. N. (1993). Music and spatial task performance. *Nature* 365 611–611. 10.1038/365611a0 8413624

[B81] Renner M. J. Lussier J. P. (2002). Environmental enrichment for the captive spectacled bear (*Tremarctos ornatus*). *Pharmacol. Biochem. Behav.* 73 279–283. 10.1016/S0091-3057(02)00786-4 12076746

[B82] Restivo L. Ferrari F. Passino E. Sgobio C. Bock J. Oostra B. A. (2005). Enriched environment promotes behavioral and morphological recovery in a mouse model for the fragile X syndrome. *Proc. Natl. Acad. Sci. U.S.A.* 102 11557–11562. 10.1073/pnas.0504984102 16076950 PMC1183589

[B83] Reynolds S. Lane S. J. Richards L. (2010). Using animal models of enriched environments to inform research on sensory integration intervention for the rehabilitation of neurodevelopmental disorders. *J. Neurodev. Disord.* 2 120–132. 10.1007/s11689-010-9053-4 22127899 PMC3164047

[B84] Reynolds S. Urruela M. Devine D. P. (2013). Effects of environmental enrichment on repetitive behaviors in the BTBR T+tf/J mouse model of autism. *Autism Res.* 6 337–343. 10.1002/aur.1298 23813950 PMC4158697

[B85] Rogers S. J. Hepburn S. Wehner E. (2003). Parent reports of sensory symptoms in toddlers with autism and those with other developmental disorders. *J. Autism Dev. Disord.* 33 631–642. 10.1023/B:JADD.0000006000.38991.a7 14714932

[B86] Rogoff B. (2003). *The Cultural Nature of Human Development.* New York, NY: Oxford University Press.

[B87] Rosenzweig M. R. (2002). Animal research on effects of experience on brain and behavior: implications for rehabilitation. *Infants Young Child.* 15 1–10. 10.1097/00001163-200210000-00003

[B88] Rosenzweig M. R. Bennett E. L. Hebert M. Morimoto H. (1978). Social grouping cannot account for cerebral effects of enriched environments. *Brain Res.* 153 563–576. 10.1016/0006-8993(78)90340-2 698794

[B89] Rueda J.-R. Ballesteros J. Tejada M.-I. (2009). Systematic review of pharmacological treatments in fragile X syndrome. *BMC Neurol.* 9:53. 10.1186/1471-2377-9-53 19822023 PMC2770029

[B90] Rusu C. Preda C. Sireteanu A. Vulpoi C. (2015). Risk factors in autism spectrum disorders: the role of genetic, epigenetic, immune and environmental interactions. *Environ. Eng. Manage. J.* 14 901–917. 10.30638/eemj.2015.101

[B91] Salami F. Ashayeri H. Estaki M. Farzad V. Entezar R. K. (2017). Studying the effectiveness of combination therapy (based on executive function and sensory integration) child-centered on the symptoms of Attention Deficit/Hyperactivity Disorder (ADHD). *Int. Educ. Stud.* 10 70–77. 10.5539/ies.v10n4p70

[B92] Sale A. Berardi N. Maffei L. (2009). Enrich the environment to empower the brain. *Trends Neurosci.* 32 233–239. 10.1016/j.tins.2008.12.004 19268375

[B93] Sale A. Berardi N. Maffei L. (2016). “Environmental enrichment and brain development,” in *Environmental Experience and Plasticity of the Developing Brain*, eds Sale A. Sale A. (Hoboken, NJ: Wiley-Blackwell), 1–26. 10.1002/9781118931684

[B94] Schneider T. Turczak J. Przewłocki R. (2006). Environmental enrichment reverses behavioral alterations in rats prenatally exposed to valproic acid: issues for a therapeutic approach in autism. *Neuropsychopharmacology* 31 36–46. 10.1038/sj.npp.1300767 15920505

[B95] Seashore C. E. (1941). The term ‘euthenics.’ *Science* 94 561–562. 10.1126/science.94.2450.561 17821263

[B96] Shaw P. Eckstrand K. Sharp W. Blumenthal J. Lerch J. P. Greenstein D. (2007). Attention-deficit/hyperactivity disorder is characterized by a delay in cortical maturation. *Proc. Natl. Acad. Sci. U.S.A.* 104 19649–19654. 10.1073/pnas.0707741104 18024590 PMC2148343

[B97] Shaw P. Gogtay N. Rapoport J. (2010). Childhood psychiatric disorders as anomalies in neurodevelopmental trajectories. *Hum. Brain Mapp.* 31 917–925. 10.1002/hbm.21028 20496382 PMC6870870

[B98] Siu A. (2014). Effectiveness of Group Theraplay^®^ on enhancing social skills among children with developmental disabilities. *Int. J. Play Ther.* 23 187–203. 10.1037/a0038158

[B99] Stoddard G. D. Wellman B. L. (1940). “Environment and the IQ,” in *The Thirty-Ninth Yearbook of the National Society for the Study of Education: Intelligence: Its Nature and Nurture, Part 1 Comparative and Critical Exposition*, ed. Whipple G. M. (Bloomington, IL: Public School Publishing Co.), 405–442.

[B100] Thomas M. S. C. Annaz D. Ansari D. Scerif G. Jarrold C. Karmiloff-Smith A. (2009). Using developmental trajectories to understand developmental disorders. *J. Speech, Lang. Hear. Res.* 52 336–358. 10.1044/1092-4388(2009/07-0144) 19252129

[B101] van Praag H. Kempermann G. Gage F. H. (1999). Running increases cell proliferation and neurogenesis in the adult mouse dentate gyrus. *Nat. Neurosci.* 2 266–270. 10.1038/6368 10195220

[B102] van Praag H. Kempermann G. Gage F. H. (2000). Neural consequences of environmental enrichment. *Nat. Rev. Neurosci.* 1 191–198. 10.1038/35044558 11257907

[B103] Varman D. R. Marimuthu G. Rajan K. E. (2012). Environmental enrichment exerts anxiolytic effects in the Indian field mouse (*Mus booduga*). *Appl. Anim. Behav. Sci.* 136 167–173. 10.1016/j.applanim.2011.12.003

[B104] Veena J. Srikumar B. N. Mahati K. Bhagya V. Raju T. R. Rao B. S. S. (2009). Enriched environment restores hippocampal cell proliferation and ameliorates cognitive deficits in chronically stressed rats. *J. Neurosci. Res.* 87 831–843. 10.1002/jnr.21907 19006089

[B105] Will B. Galani R. Kelche C. Rosenzweig M. R. (2004). Recovery from brain injury in animals: relative efficacy of environmental enrichment, physical exercise or formal training (1990-2002). *Prog. Neurobiol.* 73 167–182. 10.1016/j.pneurobio.2004.03.001 15130708

[B106] Woo C. C. Donnelly J. H. Steinberg-Epstein R. Leon M. (2015). Environmental enrichment as a therapy for autism: a clinical trial replication and extension. *Behav. Neurosci.* 129 412–422. 10.1037/bne0000068 26052790 PMC4682896

[B107] Woo C. C. Leon M. (2013). Environmental enrichment as an effective treatment for autism: a randomized controlled trial. *Behav. Neurosci.* 127 487–497. 10.1037/a0033010 23688137

[B108] Yamaguchi H. Hara Y. Ago Y. Takano E. Hasebe S. Nakazawa T. (2017). Environmental enrichment attenuates behavioral abnormalities in valproic acid-exposed autism model mice. *Behav. Brain Res.* 333 67–73. 10.1016/j.bbr.2017.06.035 28655565

[B109] Yang M. Zhodzishsky V. Crawley J. N. (2007). Social deficits in BTBR T + tf/J mice are unchanged by cross-fostering with C57BL/6J mothers. *Int. J. Dev. Neurosci.* 25 515–521. 10.1016/j.ijdevneu.2007.09.008 17980995 PMC2194756

[B110] Yochman A. Parush S. Ornoy A. (2004). Responses of preschool children with and without ADHD to sensory events in daily life. *Am. J. Occup. Ther.* 58 294–302. 10.5014/ajot.58.3.294 15202627

[B111] Zhang T.-Y. Keown C. L. Wen X. Li J. Vousden D. A. Anacker C. (2018). Environmental enrichment increases transcriptional and epigenetic differentiation between mouse dorsal and ventral dentate gyrus. *Nat. Commun.* 9 298–298. 10.1038/s41467-017-02748-x 29352183 PMC5775256

[B112] Zimmerman F. J. Christakis D. A. Meltzoff A. N. (2007). Associations between media viewing and language development in children under age 2 years. *J. Pediatr.* 151 364–368. 10.1016/j.jpeds.2007.04.071 17889070

