# Supplementary material for: Enriched Environments as a Potential Treatment for Developmental Disorders: A Critical Assessment
Source: Front Psychol. 2019 Mar 6;10:466. doi: 10.3389/fpsyg.2019.00466 (PMC6414413; doi:10.3389/fpsyg.2019.00466)
Supplement: Supplementary file 1 [file Table_1.DOCX]

*Supplementary Table 1.* (A) Animal Model Studies of Environmental Enrichment with NDDs. (B) Studies of Enrichment Treatments Involving Children with NDDs. Abbreviations: Attention Deficit Hyperactivity Disorder (ADHD), Applied Behavior Analysis (ABA), Autism Spectrum Disorder (ASD), brain-derived neurotrophic factor (BDNF), BTBR T+tf/J mice (BTBR), Developmental, Individualized, Relationship-oriented (DIR), Early Start Denver Model (ESDM), electroencephalogram (EEG), enriched environment (EE), Fragile X mental retardation 1-knock out (Fmr1-KO), Fragile X Syndrome (FXS), Pervasive Developmental Disorder-Not Otherwise Specified (PDD-NOS), postnatal day (PND), Sprague Dawley (SD), Spontaneously Hypertensive rats (SHR), valproic acid (VPA), wild type (WT), Wistar Kyoto (WKY), Wistar rats (WR).

(A)

| Study | NDD Model; animal | Grouping details; Sample Size; Age at EE exposure; Sex  *Comparison Group (Standard housing)* | Type of Enriched Environment; Duration | Relevant Findings |
| --- | --- | --- | --- | --- |
| Bechard et al. (2016) | ASD (model of repetitive behavior); deer mice | Study 1: 16 females, 10 males  EE exposed: 13 mice; PND 21  *Standard housing: 13 mice*  Study 2: 7 females, 11 males  EE exposed: 10 mice; PND 21  *Standard housing: 8 mice* | Larger housing with multiple levels, various objects including toys, tubes, and a running wheel, foraging material; 3 weeks | EE exposed mice had increased neuronal activity and dendritic spine density in the basil ganglia, and decreased repetitive behavior compared to mice in standard housing. |
| Garbugino et al. (2016) | ASD;  Oprm1 -/- knockout mice (Oprm1+/+ controls) | Enriched Oprm1 -/- : 7 litters, males and females; from birth  Enriched Oprm1+/+: 7 litters, males and females; from birth  *Standard Oprm1 -/-: 7 litters, males and females*  *Standard Oprm1+/+: 7 litters, males and females* | Extra maternal care and stimulation with two lactating mothers to one litter; birth to PND 28 | Double mothering improved the atypical response to maternal separation and increased social motivation in Oprm1 -/- mice. |
| Schneider et al. (2006) | ASD; VPA exposed in utero WR | VPA exposed (VPA-E): 24 rats; PND 7-21 and PND 22-35; males  Control (Con-E): 24 rats; PND 7-21 and PND 22-35; males  *VPA exposed (VPA): 22 rats; males*  *Control (Con): 25 rats; males* | PND 7-21: Placement of different surfaces, reflex training, negative geotaxis, and swimming; 25 minutes/day for 15 days  PND 22-35: group housing with toys | VPA-E rats had increased pain sensitivity to pain and reduced sensitivity to non-painful stimuli, increased exploratory activity and social behavior, reduced anxiety and repetitive behaviors, and stronger auditory prepulse inhibition when compared to VPA rats. |
| Reynolds et al. (2013) | ASD; BTBR mice (C57BL/6J mice as controls) | BTBR: 15 males (8 enriched mice, 7 weeks old; *7 standard housed mice*)  C57BL/6J: 16 male (8 enriched mice, 7 weeks old; *8 standard housed mice*) | More cage-mates, larger housing with multiple levels, objects including toys, tunnels, and running wheels; 30 days | Enriched BTBR mice spent less time engaging in repetitive grooming behaviors compared to standard housed BTBR mice, but the rigidity of the behavior was not improved. |
| Yamaguchi et al. (2017) | ASD; VPA exposed in utero mice | VPA exposed (VPA-E): 16 mice; 4 weeks old; males  Saline exposed (SE-E): 16 mice; 4 weeks old; males  *VPA exposed (VPA): 16 mice; males*  *Saline exposed (SE): 16 mice; males* | Larger housing, running wheels, different nesting materials, objects for play and exploration; 4 weeks | Enriched environment exposed mice had increased BDNF compared to mice in standard housing. The VPA-E group showed improved social and cognitive deficits and anxiety, and partial rescue of hippocampal dendritic spines compared to the VPA group. |
| Yang et al. (2007) | ASD; BTBR mice (C57BL/6J mice as controls) | BTBR pups: *Own mother,* different BTBR mother, C57BL/6J mother  C57BL/6J pups: *Own mother*, different C57BL/6J mother, BTBR mother  8-12 pups in each condition; pups placed with foster mothers within 24 hours of birth; males and females | Cross-mothering from foster C57BL/6J mother; 21 $\pm$1 days | BTBR pups fostered by more sociable C57BL/6J mothers did not show improvements in play and sociability nor any reductions in grooming behaviors. Additionally, C57BL/6J pups fostered by BTBR mothers did not show deficits in play. |
|  |  |  |  |  |
| Botanas et al. (2016) | ADHD; SHR (WKY as controls) | SHR-EE: PND 21; males  WKY-EE: PND 21; males  *SHR-standard housing:* PND 21; males  *WKY-standard housing:* PND 21; males | Larger cages, toys, shelter, tubes, and a running wheel; PND 21-49 | Enriched environment exposed rats shows improved performance in Y-maze (measure of inattention) and open-field test, but not delay discounting task (measure of impulsivity). EEG changes were found in the WKY-EE controls, but not SHR-EE. |
| Gauthier et al. (2015) | ADHD; SHR (WKY as controls) | SHR pups: *SHR mother (4 dams),* or WKY mother (5 dams)  WKY pups: *WKY mother (8 dams)*, or SHR mother (7 dams)  3-4 pups per litter; pups placed with foster mother on PND 1 | Cross fostering from WKY mother; PND 1-25 | Hyperactivity was dependent on pup strain alone. Social behavior was dependent on strain of the mother, while attention orientation was affected by both strain of pups and foster mother. Anxiety was affected by an interaction between pup and foster mother strain. |
| Howells et al. (2009) | ADHD; SHR (WKY and SD as controls) | SHR pups: SHR mother, WKY mother, or SD mother  *WKY pups: WKY mother, SHR mother, or SD mother*  *SD pups: SD mother, SHR mother, or WKY mother*  10 to 15 pups per group, 5 to 6 litters,  2 to 4 rats from each litter tested | Cross fostering from WKY or SD mother; PND 2-21 | SHRs raised by WKY or SD mothers did not show differences in open field or elevated plus- maze or hippocampal and prefrontal cortex neurochemistry compared to control pups raised by SHR mothers. |
| Pamplona et al. (2009) | ADHD; SHR (WR as controls) | SHR-EE; PND 21; males  WR-EE; PND 21; males  *SHR-Standard housing*  *WR-Standard housing* | More cage-mates, larger housing, objects including toys, tubes, and a running wheel; PND 21 to 3 months | SHR-EE rats showed improved performance in open field habituation, recognition tasks, and water maze tasks. EE did not affect control rat performance. |
|  |  |  |  |  |
| Oddi et al. (2015) | FXS; Fmr1-KO mice (WT mice as controls) | Fmr1-KO-EE; males  WT-EE; males  *Fmr1-KO-Standard housing*; males  *WT-Standard housing*; males  6 to 11 offspring from each housing condition were used testing, with 3 cohorts of offspring for each testing group | Lactating mother plus a non-lactating NMRI virgin female until PND 21 | Enriched Fmr1-KO pups showed reduction in hyperactivity and improvement in social and cognitive deficits, as well as rescue of abnormalities in the hippocampus and amygdala. Enrichment did not have similar effects on neurology or behavior in WT mice. |
| Restivo et al. (2005) | FXS; Fmr1-KO mice (WT mice as controls) | Fmr1-KO-EE; 9 mice; PND 21; males  WT-EE; 9 mice; PND 21; males  *Fmr1-KO-Standard housing; 9 mice; PND 21; males*  *WT-Standard housing; 9 mice; PND 21;* *males* | Larger cages with multiple levels, toys (boxes, tubes, balls, bells), running wheel, nesting material, and 2hr/day additional enriched environment with foam, iron objects, and boxes; 60 days | Enriched Fmr1-KO mice showed amelioration of anxiety-like behavior in open field task, restored habituation to objects, and improved dendritic morphology in visual cortex. GluR1 levels were increased in both enriched genotypes. |

(B)

| Study | NDD Focus; Type of Intervention | Grouping details; Sample Size; Age at EE exposure; Sex  *Comparison Group* | Type of Enrichment; Duration | Relevant Findings |
| --- | --- | --- | --- | --- |
| Aronoff et al. (2016) | ASD; Sensory Enrichment Therapy | 1,002 children (varying in geographic location, payment for service, and formal diagnosis); age 1-18 years; 206 females, 796 males | Customized program based on behavioral symptoms given to parents via Mendability, LCC, an online service. Tactile, thermal, visual, auditory, vestibular and proprioceptive, and olfactory enrichment exercises delivered daily by parents for 1-6+ months | Based on parent assessment, enriched children showed improvements in learning, memory and attention, motor skills, sleeping and eating, sensory processing, communication and social skills, and anxiety, mood, and autism-related behaviors. |
| Dawson et al. (2010) | ASD; ESDM | ESDM: 24 children with ASD; 18-30 months of age; 3.5:1 male-to-female ratio  *Control (other community-based intervention): 24 children with ASD; 18-30 months of age; 3.5:1 male-to-female ratio* | ESDM strategies and techniques including: adult responsivity to child’s cues with positive affect, focus on communication, and teaching strategies consistent with ABA implemented by trained parents and therapists; ~25 hours a week for 2 years | Children who received ESDM treatment showed improvements in IQ, adaptive behaviors, and autism diagnosis compared to controls. |
| Hiles Howard et al. (2017) | ASD; Theraplay | Theraplay: 8 children with ASD; 3-9 years of age; 2 females, 6 males | Child and parent dyad had sessions with a trained therapist, focusing on nonverbal communication, child regulation, and parent sensitivity using play as a medium; 2 1-hour sessions per day for 9 days | Both children and parents improved at interacting during play sessions over time, with parents showing better responsiveness, eye contact, and guidance with their child, and children increasing eye contact, vocalization, closer proximity to parent, and acceptance of guidance. |
| Pajareya & Nopmaneejumruslers, (2011) | ASD; (DIR)/Floortime™ | (DIR)/Floortime™: 16 children with ASD or PDD-NOS; ages 24-72 months of age; 8:1 male-to-female ratio  *Control (Typical treatment): 16 children with autism or PDD-NOS; ages 24-72 months of age; 8:1 male-to-female ratio* | Experimental group parents were trained on how to use (DIR)/Floortime™ at home, aimed at improving communication and focusing on positive parent-child interactions; average of 15.2 hours a week for 3 months | Children who received (DIR)/Floortime™ treatment showed improvements on functional emotional scales and autism symptom severity compared to controls. |
| Pajareya & Nopmaneejumruslers, (2012) | ASD; (DIR)/Floortime™ | 34 children with ASD or PDD-NOS; age 2-6 years; 7.5:1 male-to-female ratio | Experimental group parents were trained on how to use (DIR)/Floortime™ at home, aimed at improving communication and focusing on positive parent-child interactions; average of 14.2 hours per week for 12 months | Children with less severe autism benefited more from treatment. |
| Woo et al. (2015) | ASD; sensorimotor enrichment | Enriched group: 28 children with ASD, age 3-6 years; 5 females, 23 males  *Standard care group: 22 children with ASD, age 3-6 years; 2 females, 20 males* | Tactile, olfactory, auditory, thermal, visual, and motor enrichment, including music enrichment and sensorimotor exercises delivered by parents; daily for 6 months | Enriched children showed increases in IQ scores and receptive language performance, and reduction of abnormal sensory responses compared to controls. |
| Woo & Leon, (2013) | ASD; sensorimotor enrichment | Enriched group: 13 children with ASD, age 3-12 years; males  *Standard care group: 15 children with ASD, age 3-12 years; males* | Tactile, olfactory, auditory, thermal, visual, and motor enrichment, including music enrichment and sensorimotor exercises delivered by parents; daily for 6 months | Enriched children showed decreases in severity of ASD, and improvements in cognition task scores relative to controls. |
|  |  |  |  |  |
| Salami (2017) | ADHD; Combination therapy (Executive Function and Sensory Stimulation) | Treatment group: 20 children with ADHD; 1^st^, 2^nd^, and 3^rd^ graders  Control group (no treatment): 20 children with ADHD; 1^st^, 2^nd^, and 3^rd^ graders | Combination therapy consisted of activities to increase concentration, working memory, and behavior inhibition, and tactile and deep system stimulation; 24 1-hour sessions, 3x/week for 2 months (in groups of 5 children) | Children in the treatment group showed lower scores on attention deficiency and hyperactivity compared to controls. |
